# Changing gender norms around female genital mutilation/cutting (FGM/C): a key role for social work in the Global North

**DOI:** 10.3389/fsoc.2023.1187981

**Published:** 2023-06-02

**Authors:** Michela Villani

**Affiliations:** School of Social Work, University of Applied Sciences and Arts Western Switzerland (HES-SO), Fribourg, Switzerland

**Keywords:** female genital mutilation/cutting (FGM/C), social work, social norms, sexuality, gender equality, Global North

## Abstract

“Female genital mutilation/cutting” (FGM/C) refers to procedures that involve altering the external female genitalia with the aim of reinforcing gendered body norms. The literature has consistently shown that, like various forms of discrimination, the practice is rooted in systems of gender inequality. As a result, FGM/C has increasingly come to be understood in terms of social norms that are by no means fixed. And yet, in the Global North, interventions remain primarily medical in nature, with clitoral reconstruction having emerged as a common means of dealing with related sexual issues. And although treatments can vary greatly depending on the hospitals and physicians involved, sexuality tends to be considered from a gynecological perspective, even when multidisciplinary care is offered. By contrast, gender norms and other socio-cultural factors receive little attention. In addition to highlighting three critical shortcomings in current responses to FGM/C, this literature review also describes how social work can play a key role in overcoming the associated barriers by (1) adopting a holistic approach to sex education, one capable of addressing those aspects of sexuality that lie beyond the scope of a medical consultation; (2) supporting family-based discussions on matters of sexuality; and (3) promoting gender equality, especially among younger generations.

## 1. Introduction

“Female genital mutilation/cutting” (FGM/C) refers to procedures that involve altering the external female genitalia by cutting the clitoris and/or labia, or by narrowing the vaginal orifice (WHO, 2008). And although the term does not normally encompass procedures like labia minora reduction (nymphoplasty), genital cosmetic surgery is being performed on growing numbers of young women, including minors (Kalampalikis and Michala, 2021). This has led to growing calls to analyze FGM/C and genital cosmetic surgery in parallel, insofar as both sets of practices are rooted in systems of gender inequality (Ziyada et al., 2020; O'Neill et al., 2021; Villani, 2022) and reinforce certain gendered body norms that associate femininity with slenderness, diminutiveness, docility, reduction, etc. Through their interactions with affected populations (youth, migrants), social workers are regularly confronted with issues of sexuality and gender. In this context, promoting egalitarian gender norms, raising awareness of sexual rights, and addressing sexual wellbeing (including as a means of preventing sexual violence) are all more or less central to the mission of social work.

Worldwide, about 200 million women and girls have undergone some form of FGM/C (UNICEF, 2016). Traditionally practiced in Africa, the Middle East, and Asia, it has spread to Europe and other parts of the world (UNICEF, 2016). Meanwhile, bans introduced in many European and African countries have failed to adequately curtail the practice (Boyle and Cotton Corl, 2010). Research shows that although legal restrictions have proven more effective in the Global North, the success of law enforcement efforts often depends on the presence of more informal social control mechanisms (Boyle and Cotton Corl, 2010). In other words, in the absence of awareness campaigns, media coverage, family discussions, and other informal measures, so-called anti-FGM laws risk further harm to migrants from sub-Saharan Africa by reinforcing existing forms of discrimination or producing new ones (O'Neill et al., 2020). Anti-FGM awareness campaigns have also been accused of exposing women to increased stigma by adopting colonial attitudes toward the populations concerned (Manderson, 2004; Njambi, 2004; Boddy, 2016). In some African communities, the backlash against such campaigns has even led to a resurgence of FGM/C, especially where the message was perceived as racist or discriminatory (La Barbera, 2009; Graamans et al., 2019). As a result, development programs have begun to scrutinize how they address FGM/C when conducting outreach to indigenous populations (UNFPA, 2014).

In keeping with a medicalized approach to FGM/C, awareness campaigns tend to emphasize health concerns. Advocates can draw on a large body of medical literature on the adverse effects of FGM/C, including its impacts on obstetrical, gynecological, psychological, and sexual health (Berg et al., 2010, 2014). By comparison, a gender-based interpretation that emphasizes the fundamentally discriminatory dimension of the practice has been slow to take hold at the international level (WHO, 2008). Nevertheless, FGM/C has increasingly come to be understood in terms of intergenerationally transmitted (Berg and Denison, 2013; Boyle and Svec, 2019) social norms rooted in gender inequality (Ziyada et al., 2020; O'Neill et al., 2021; Villani, 2022). Applied research focused on the relationship between social norms and FGM/C (Mackie et al., 2015) has determined that they are by no means fixed (Mackie and Le Jeune, 2009). Tools have even been developed to help change attitudes and foster discussion (Lennie and Tacchi, 2013), especially using videos and other media (Vogt et al., 2016; Villani et al., 2019b). However, in the Global North, medical specialists remain those most likely to encounter women and girls who have undergone FGM/C. Often with very little preparation, these professionals find themselves forced to navigate a range of factors involving cultural traditions, gender norms, and sexuality (Berggren et al., 2006; Jacobson et al., 2018). In addition, financial constraints within the healthcare sector often reduce the time available to discuss such issues. Against this backdrop, the field of social work could certainly provide vital support to hospitals, schools, and associations through a yet-to-be-defined model of interprofessional collaboration.

A holistic approach to sex education would emphasize the multifaceted nature of sexuality, highlighting its many interconnected dimensions (biological, medical, emotional, social, legal, etc.). However, special attention would be paid to sexual rights. Naturally, this would involve the transmission of knowledge. But it would also require a commitment to improving the social and psychosocial skills of individuals with a view to having an active role played by both the family and the network of health and social work professionals. Given the range of populations involved (migrants, persons seeking assistance, persons with disabilities, children and adolescents, victims of violence, etc.), social workers would appear to constitute the professional group best equipped to support and engage families in the sex education process. Accordingly, this article strongly emphasizes the potential role for social work professionals, highlighting the new frontiers and perspectives they could explore.

Based on a thorough review of the literature, this article identifies three significant shortcomings that the field of social work could help address. I begin by highlighting the need to take a less medicalized approach to FGM/C and sexuality by addressing health concerns alongside other factors better dealt with outside a hospital setting. Second, I address the paradox stemming from a binary opposition between the “private” (family values transmitted by parents responsible for their children's sexual education) and the “public” (social norms related to sexuality transmitted by health and social professionals) by exploring inequalities from the perspective of a family's origins and cultural circumstances. Finally, the third section of the article considers notions of gender equality, consent, and sexual self-determination in terms of the need to foster democratic and participatory forums for discussing sexual and gender norms.

## 2. Shortcomings in responses to FGM/C

With the aim of identifying shortcomings in responses to FGM/C, I undertake a purposive literature review with a focus on articles that have analyzed the practice in relation to gender and sexual norms. Based on their potential to help define a larger role for social work, I primarily discuss studies that analyze responses to FGM/C in diasporic contexts (i.e., in the Global North). Nevertheless, the critical discussion presented below also draws on some recent work undertaken in the Global South to better contextualize the challenges faced as well as the ways in which social work could help meet them. And recognizing the similarities between social work and development cooperation efforts, I draw some parallels between experiences in the two domains.

**Figure 1:**
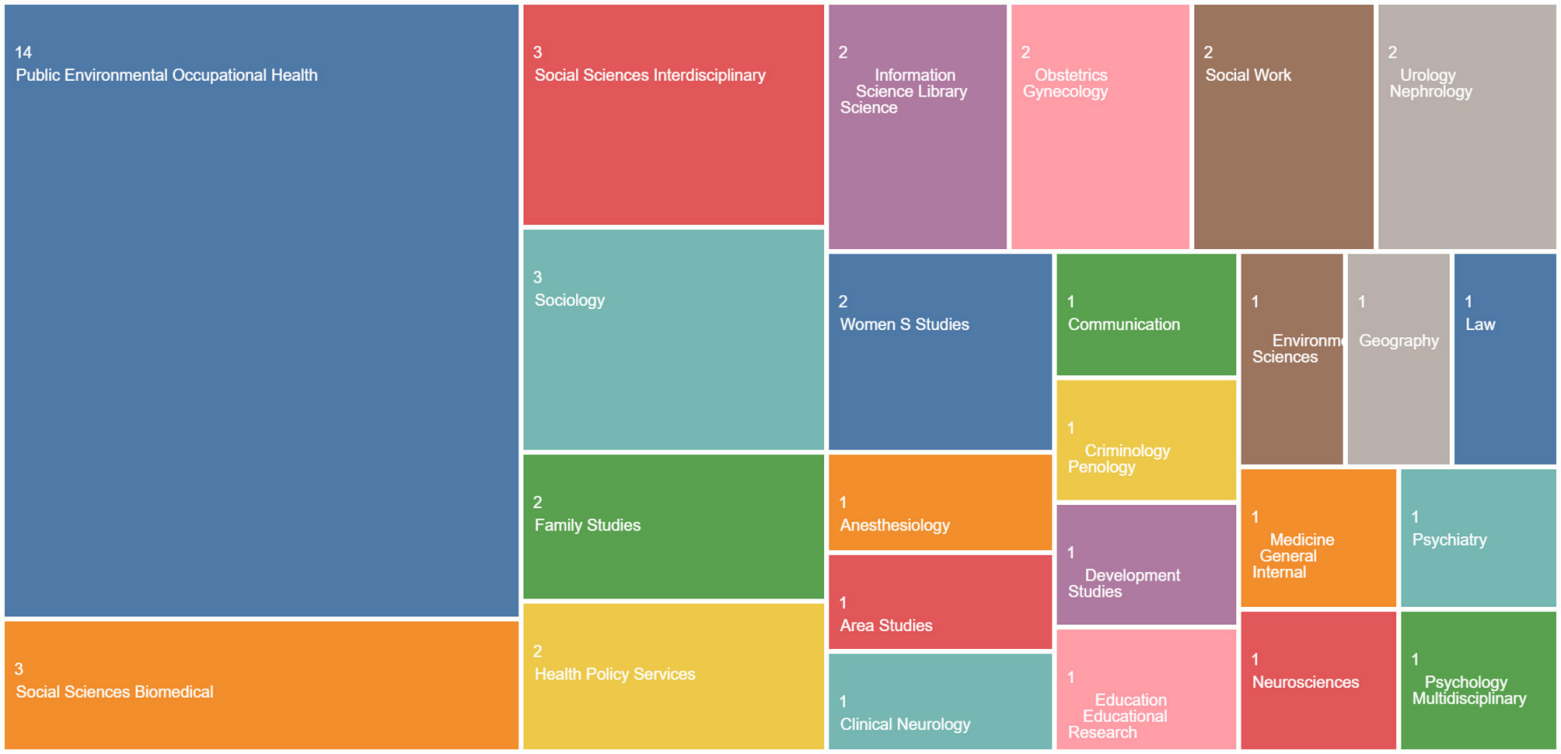
Body of texts analyzed on the Web of Science platform (Diagram generated by © 2023 Clarivate).

I primarily relied on the Web of Science platform to establish the body of texts to be reviewed. I began by searching for the terms “female genital mutilation” (Topic) and “social work” (All Fields) in peer-reviewed articles published in English. I went on to review the 111 results, retaining articles dealing with the relationship between FGM/C and social or health interventions, and setting aside those focused on surgical techniques or gynecological issues. Nevertheless, several examples of medical literature were retained insofar as they addressed sociocultural issues and/or question of legal and medical ethics. Seeking out interdisciplinary approaches, I selected a final set of 31 articles published in journals reflecting a range of disciplinary perspectives. Falling under the category of Public Environmental Occupational Health, 45% of the retained articles directly analyzed social and health interventions related to FGM/C. Overall, I considered 16 examples of social science and policy research or applied research in education, communication, etc.; two studies in the field of law and criminology; and 13 articles from various branches of medical research. Figure 1 illustrates the distribution of articles and journals by discipline.

### 2.1. Dispelling myths around sexuality through a holistic approach to sex education

Medical scholars have produced a significant number of articles on FGM/C. This work has largely focused on the impact of the practice on the reproductive, sexual, and psychological health of women and girls (Berg et al., 2010, 2014). More recent examples have shifted attention to reconstructive surgical techniques (Abdulcadir et al., 2015) and postoperative complications (Bah et al., 2021), as well as the potential role of counseling and multidisciplinary support in treating individuals who have undergone FGM/C (De Schrijver et al., 2016). By contrast, little has been written on surgical outcomes, pain management, and self-image. Likewise, the limited research on sexual pleasure has failed to reach any clear conclusions. Several studies have emphasized the difficulty of establishing direct links between FGM/C and the presence or absence of sexual pleasure (Obermeyer, 2005; Catania et al., 2007), given the need to consider other related factors, including self-image, relationship quality, and social stigma (Palm et al., 2019; Jordal et al., 2022). On the one hand, the medicalization of sexuality has produced a rather narrow approach to assessing sexual health (Giami, 2002), one that minimizes the social dimension and obscures the role played by socialization processes (Gagnon, 1990; Longmore, 1998). As a result, the professionals most often called upon to deal with issues of sexuality work in the medical field: gynecologists, nurses, midwives, psychologists, and sexologists. On the other hand, efforts to achieve a more complex understanding of “sexual health” (Giami, 2002) have led to a recognition of the multidimensional nature of sexuality. Accordingly, the World Health Organization (WHO, 2006) has set about developing a holistic or comprehensive approach to sex education that considers a wide range of factors: biological, medical, emotional, social, legal, etc. Such an approach allows for a simultaneous focus on sexual rights and the development of social and psychosocial skills (Charmillot and Jacot-Descombes, 2018).

But in practice, health professionals often serve as the first point of contact for those seeking help with FGM/C-related issues, and this is especially true for migrants. Some researchers have noted how “primary healthcare professionals are ideally placed to detect and prevent these situations of risk” (Kaplan-Marcusán et al., 2010). Others have emphasized the various difficulties that arise when working with migrants and the associated barriers (linguistic, material, legal, etc.) to care (Asanin and Wilson, 2008). A third group of studies have looked at the attitudes of health professionals toward clitoral reconstruction (Jordal et al., 2021) and caregivers, as well as how cultural bias shapes the therapeutic relationship alongside social and media constructions of FGM/C (Palm et al., 2019). For instance, some articles have emphasized the need for health and social professionals to suspend judgment and actively reflect on their own feelings, reactions, values, and beliefs (Onsongo, 2017). To begin with, such a process can help avoid binary oppositions between freedom and oppression, us and them, civilization and barbarism, etc. Furthermore, many studies have noted the important role of community workers in facilitating access to care. Not only do medical professionals encounter pervasive cultural and gender issues when treating individuals having undergone FGM/C, but they are also at increased risk of making overly culturalist interpretations of the practice when addressing matters of sexuality.

With these points in mind, some scholars have identified an underlying medical bias in how sexual issues are treated in women who have undergone FGM/C (Earp, 2020). A reductive focus on the role of genitalia and a neglect of other important dimensions of sexuality can negatively impact a woman's body image and sexual self-esteem. The studies in question have recommended avoiding comments that could cause feelings of body shame or imply a loss of sexual capacity (Villani, 2015; Palm et al., 2019). Other scholars have explored the emotional burden shouldered by women who have undergone FGM/C. For instance, one recent study notes the importance of not only providing such women with anatomical knowledge but also dispelling myths and combatting misinformation about female sexual anatomy and physiology (Abdulcadir et al., 2020). Although this task may appear too formidable and important to carry out in the context of an ordinary appointment with a gynecologist, addressing such topics in a comprehensive and inclusive manner appears essential if professionals are to avoid reproducing heteronormative and cissexist views, which represent a frequent source of medical bias (Payne and Smith, 2016). Examples of important themes that such biases have caused to be overlooked in the literature include the sexuality of lesbians who have undergone FGM/C.

More recent studies dealing with clitoral reconstruction have explored the relationship between stigma and gender norms (O'Neill et al., 2021; Jordal et al., 2022; Villani, 2022) by analyzing the impact of migration on the perception of self as a “mutilated” woman (Johnsdotter and Essén, 2016). For their part, postcolonial feminist critiques have highlighted how “othering” processes racialize those who have undergone FGM/C. For instance, Black women, even those born or raised in Europe, are ascribed an Africanness that serves to associate the practice with the “other” (Ahmadu and Shweder, 2009; Villani, 2015). The difficulty of framing FGM/C in a way that avoids the pitfalls often associated with discourses of cultural relativism and universal human rights (Evans, 2020) has led researchers to treat the practice as part of a broader continuum (Fusaschi, 2023). Drawing parallels with other interventions (male circumcision, intersex surgery, labia minora reduction, etc.) that alter genitalia in ways designed to reinforce the male/female dichotomy can open new avenues for reflection (Earp et al., 2023). Finally, issues around clitoral reconstruction serve to highlight the need for a multidisciplinary and holistic approach to genital alteration as it is practiced in the Global North (Villani, 2015; De Schrijver et al., 2016; Jordal and Griffin, 2018).

Medical consultations can give rise to discussions on largely non-medical themes, including issues of morality (FGM/C as a bad practice), ethics (reporting the parents, suggesting reconstruction, etc.), and ideology (humanitarianism, feminism, other forms of activism). Such discussions require significant emotional effort on the part of patients as they come to terms with the therapeutic relationship (Jacobson et al., 2022). Some authors have argued that international migration contributes to a clash between traditional practices and the host society's cross-cultural imperative within care systems struggling to reconcile the two positions (Vissandjée et al., 2014). In the course of their work, medical specialists are called upon to perform the ethically complex task of setting aside their own identity and culture, which often conflict with the identities and cultural processes of the migrants they are expected to assist, while also remaining bound by legal and professional constraints. Other researchers have pointed out how the promotion of cross-cultural policies runs the risk of reinforcing “the hegemonic nature of the patient-practitioner interactions and politics of Otherness” (Koukoui, 2019).

This raises various ethical questions, including whether physicians should seek to “dispel” mistaken beliefs about sexuality and, if so, in what therapeutic setting. Furthermore, where is the line between accurate and harmful beliefs, and how can it be drawn in a way that respects individual freedom? Finally, what tools do hospital departments have at their disposal for addressing such questions and the various impacts of myths, beliefs, and social norms on sexuality—all in the context of very brief appointments? At the same time, the physician's position as an authority figure is problematic, insofar as the doctor-patient relationship is defined by an imbalance of power. In any case, sexual norms are transmitted by a range of actors—including relatives (intergenerational and transgenerational transmission)—meaning that, in the context of a medical consultation, an intervention focused on the individual can only go so far in changing attitudes, social norms, or even beliefs.

By contrast, a holistic approach to sex education offers other ways of addressing these different factors. For instance, social workers are well positioned to sidestep medical bias and a potentially normative reading of sexuality. Looking at the European context, Charmillot and Jacot-Descombes (2018) have shown how such professionals can contribute to sex education through their family support efforts. However, barriers remain; in many countries, social work training programs fail to address sexuality in any meaningful way (Rowntree, 2014; Carbajal and Colombo, 2021). The resulting lack of knowledge means that, in practice, social workers rarely address questions of sexuality (Schaub et al., 2017). Other authors have shown how, to the extent that social work education programs address such issues, they tend to discuss sexual minorities in negative terms and focus on overt expressions of homophobia (Morton et al., 2013). Such studies underscore the pressing need to strengthen the field's capacity for providing critical insights and to systematically address sexuality in the basic social work curriculum (McCave et al., 2014), with the aim of developing a more holistic approach to sex education (Dodd and Tolman, 2017).

In this context, social work can play a vital role, especially among younger generations. Participatory outreach activities and preventive interventions could be designed to promote cross-cultural dialogue and ensure respect for diverse cultural identities. For example, outreach or even educational activities could draw connections between FGM/C and various other issues (obstetrical and gynecological violence, sexual violence, etc.) that can impact the sexual lives of women who may seek out treatment or reconstructive care.

### 2.2. Private vs. public: how health and social work professionals can support family discussions on sexuality by challenging gender norms

Communication-focused studies on FGM/C have highlighted the difficulty of addressing related issues in a family context. The taboo around sex reflects not only a lack of comfort in discussing sexuality but also “pluralistic ignorance” (Mackie et al., 2015). In the field of interactionist sociology, this term describes situations where members of a group or community consistently support a dominant social norm even though many of them question its validity. Convinced that they are the only ones who think differently, individual skeptics avoid expressing dissent or even conferring with others. As a result, the social norm remains entrenched and unchallenged. This helps explain why FGM/C, when practiced out of cultural conformity, acquires a social significance: it can express a sense of identity and respectability as a member in good standing of the community. In its context of origin, this meaningful cultural tradition represents a form of social and identity control for women, as well as a distinguishing feature of the ideal girl (Shell-Duncan et al., 2021). The practice is deeply rooted in social systems and its mandatory nature is reflected in the community mechanisms used to enforce it (social acceptance and exclusion). At the community level, efforts to enforce tradition strongly rely on notions of honor and a desire to avoid shame on the part of not only daughters but also mothers and sometimes the extended family (Shell-Duncan et al., 2021). Accordingly, it would seem all the more important to promote dialogue and support family discussions on sexuality that involve adolescents (WHO, 2018).

With respect to cultural factors that affect how individuals are socialized to talk about sex, a study of first- and second-generation youth in Switzerland with roots in sub-Saharan Africa has highlighted the difficulties they face when trying to discuss sexuality with their parents. For members of the second generation, sexuality socialization involves dealing with socio-cultural barriers as well as conflicting social norms. For example, broaching the topic of sexuality with an older person can be interpreted as a sign of “disrespect” (Sulstarova et al., 2019). As for parents, they tend to avoid the topic for fear of “encouraging” youth sexual activity (Sulstarova et al., 2019). Accordingly, families consistently avoid conversations about sexuality under the terms of a tacit agreement acknowledging sex as a taboo subject that should only be addressed “in secret” (Villani et al., 2019a). Studies have also found that first-generation migrant youth in Switzerland lack knowledge of sexual rights and available sexual health services (Campisi et al., 2017; Mileti et al., 2019). This further underscores the importance of focusing on risk behaviors among adolescents as they discover their sexuality (Barrense-Dias et al., 2022).

Other studies have noted how social and physiological events can serve as “triggers” to initiate discussion (Moulin, 2007). But although concerns about the personal, social, and economic consequences of risky sexual behavior (teen pregnancy, abortion, STIs, etc.) can spark conversations, they can also discourage openness. Coupled with cultural and religious norms, parents' inability to effectively address issues of sexuality creates an uncomfortable environment—makes peers, the media, teachers, and siblings leading and sometimes preferred sources of sexual health information (Usonwu et al., 2021). Researchers have also found that conversations with parents about sexuality tend to be authoritarian and one-directional, characterized by vague warnings rather than direct and open discussion. Ultimately, a lack of open dialogue between parents and youth, a lack of knowledge and skills, and cultural norms and taboos all compound the situation (Bastien et al., 2011). Furthermore, linguistic, cultural, and symbolic barriers can stand in the way of discussions on sex (Sulstarova et al., 2019), significantly hindering communication between parents and children in various ethnocultural contexts in both the Global South and the Global North (Mellini and Poglia Mileti, 2021).

It follows that FGM/C is rarely discussed in family contexts and very little is said to girls before they undergo the practice. At very least, diasporic women consistently report this as having been their experience (Andro et al., 2010; Villani et al., 2016). Indeed, although FGM/C can take on a significant social and ritual dimension by marking the transition to adulthood, it would seem to involve little in the way of knowledge transfer. It is often planned without the knowledge of the person most directly concerned (Andro et al., 2010; Villani et al., 2016), and even by way of deception. This is especially true in cases where a relative organizes FGM/C without the knowledge of one (or both) parents. Granted, the community aspect may still be present in some contexts. However, FGM/C is increasingly devoid of any ritual dimension. Rather, it tends to be carried out at a young age, in a private or even medicalized setting (Kimani and Shell-Duncan, 2018). Women rarely have good memories of the procedure (Andro et al., 2010; Villani et al., 2016) and often stress the importance of “breaking the silence” within families when sharing their experiences (Andro et al., 2010). At the same time, feelings of guilt (on the part of parents) and a sense of loyalty (on the part of those who undergo FGM/C) help maintain a code of silence in relation to the practice (Villani and Bodenmann, 2017). In a diasporic context, abandoning FGM/C can be seen as a severing of ties or even a betrayal. As a result, parents who oppose the practice in principle may give in to pressure and have FGM/C performed on their daughter to “please the family” (Andro et al., 2010). In some cases, the clash between the sexual norms of the host country and those of the country of origin can lead to intergenerational conflict within families. A study involving migrant communities in the Global North has found that belief systems originating in the Global South are not only able to persist in a diasporic context but remain largely unchanged (Alhassan et al., 2016). A better understanding of how sexual norms shift within migrant families—whether reflected in the rejection of GFM/C or the abandonment of traditional values (regarding virginity, marriage, homosexuality, etc.)—could be achieved through a focus on intergenerational transmission. Such a perspective would allow for reassessing the symbolic significance of abandoning or altering a tradition, as well as the impact on the sense of belonging (Johansen and Ahmed, 2021).

A UK-based study involving foreign-born women who had undergone FGM/C has found that although the research participants expressed opposition to the practice and sought to abandon it, none of them had reported their experiences to the police. They also explained that even if they discovered that FGM/C was being performed in their communities, they would not report it to the police because they did not trust the authorities. These women saw community-based efforts as being more effective than turning to the criminal justice system (Gangoli et al., 2018). Other researchers have found that girls are less likely to undergo FGM/C when their parents take a joint approach to making major household decisions. This provides new insights into how women, families, and communities can disrupt the intergenerational transmission of behaviors rooted in institutionalized forms of gender discrimination (Boyle and Svec, 2019). They also highlight the need to support family discussions on sexuality. Finally, a study conducted in the United States and involving individuals at risk of undergoing or having undergone FGM/C has noted the lack of tools and knowledge available to those health and social professionals called upon to work with such populations. Even professionals with a basic knowledge of the issues at play expressed uncertainty about the current legality of FGM/C in the United States and described a lack of training related to the practice (Akinsulure-Smith et al., 2021).

In a cross-cultural context, promoting and facilitating family discussions on sexuality and FGM/C is of utmost importance. The literature shows how, in diasporic communities, the clash between the sexual norms embraced by parents (which often reflect those prevalent in the country of origin) and those adopted by their children (which are more likely to reflect norms prevalent in the host country) can be a source of intergenerational conflict. Social workers could be tasked with developing intervention strategies that make it possible to address these factors while exploring experiences of change (in cultural values, in cultural norms) and even grief (related to the permanent abandonment of certain cultural traditions). For example, parents could be supported through a mix of cross-cultural mediation and collaboration with resource people based in local ethnocultural communities. In what is no doubt a reflection of the traditional healthcare-based approach to sexuality, social professionals have described feelings of “illegitimacy” when seeking to deal with sexual issues (Carbajal and Colombo, 2021). However, a healthcare-based medical approach remains inadequate for addressing the complex issues involved, which extend far beyond purely medical concerns. Clearly, the social dimension needs to be central to both prevention efforts and relationships with individuals or communities.

### 2.3. Promoting gender equality and sexual rights among youth by emphasizing self-determination and autonomy

Various considerations justify a focus on younger generations. To begin with, young people (especially those under the age of majority) enjoy special legal protections, which vary from one country to the next. For instance, jurisdictions design sex education programs and harm prevention efforts in relation to a legal age of consent, generally set at 16. Such initiatives address the risks associated with sexually transmitted infections (STIs) and teen pregnancy, as well as sexual violence and abuse. Meanwhile, adopting a holistic approach to sex education would emphasize the concepts of sexual rights and sexual well-being, which together help communicate the notion of “sexual self-determination.” In this regard, the document titled *Sexual rights: An IPPF declaration* (International Planned Parenthood Federation, 2008) constitutes an essential tool for organizations, activists, researchers, and decision-makers working to promote and secure human rights. Specifically, Article 5 refers to a person's right to autonomy in terms of exploring their emotional and sexual orientation, as well as their gender identity.

In matters of sexuality, the promotion of gender equality, especially among younger generations, should certainly represent a major objective. But whereas signatory countries to the Istanbul Convention (Council of Europe, 2011) have committed to this principle, gender-based social expectations serve to reproduce structural inequalities that shape how young people discover their sexuality (Clair, 2008) and negotiate sexual relationships (Colombo et al., 2017). Accordingly, when their work is viewed through the lens of “gender policing” (Payne and Smith, 2016), health and social professionals are at risk of reinforcing gender stereotypes and socially prescribed forms of sexual behavior. From this perspective, training is crucial to ensuring that professionals can effectively support youth in learning about sex while protecting them from infringements of their sexual rights (abuse, violence, etc.) (Carbajal and Colombo, 2021). Likewise, the media play an important role in reinforcing or challenging gender-based notions of sexuality (Chmielewski et al., 2017).

Populations that practice FGM/C are by no means the only ones concerned by the issue of equality. Rather, gender discrimination cuts across a wide range of ethnocultural contexts. For their part, Western societies have traditionally promoted gender models characterized by inequality and male domination (Collins, 1991; Hooks, 1992; Scott, 2006). FGM/C can therefore be seen as the expression of “historically unequal power relations between women and men, which have led to domination over, and discrimination against, women by men and to the prevention of the full advancement of women” (Council of Europe, 2011), alongside various other forms of sexual and gender-based violence. Such violence is rooted in an inherent desire to dominate women as a gender by restricting their freedom to act and their sexual autonomy. Accordingly, the legally binding Istanbul Convention is based on the idea that “the realization of de jure and de facto equality between women and men is a key element in the prevention of violence against women”(Council of Europe, 2011).

A comprehensive literature review has identified six key factors underlying FGM/C: cultural tradition, sexual morals, marriageability, religion, health benefits, and male sexual enjoyment (Berg and Denison, 2013). Social pressure to conform also plays a key role. A recent study involving 1,400 youth between the ages of 15 and 25 has examined the meanings associated with FGM/C in three distinct indigenous contexts in Indonesia, Ethiopia, and Kenya (Kakal et al., 2022). The results confirm the conclusions of earlier studies by showing how the pursuit of a bodily ideal (pure, clean) and sexual morality (limited libido) remains constant. In other words, FGM/C serves to “tame” the body of a future woman, giving it a form considered suitable for marriage. In this respect, the practice is rooted in gender norms that dictate how young women should express their femininity by embodying specific characteristics and adopting traditional roles. The female body is the medium through which these norms are negotiated. FGM/C alters the “natural body” into a “cultural body,” in the same way as Western practices of body transformation or genital modification (Fusaschi, 2023). For instance, among the Maasai, FGM/C is seen as creating an “adult body” that allows young women to begin behaving like adults (Kakal et al., 2022). Furthermore, social norms around gender roles also shape male behavior and the construction of masculinity (Connell, 2005). Any attempt to revise the associated gendered social norms will therefore require identifying the underlying representations that will trigger resistance to change (Bicchieri, 2016). It is also important for transformative rituals, whose development could be fostered by social workers using mediation and support tools, to continue framing the coming-of-age process as well as social roles.

Multiple studies have sought to determine the best approaches to communicating with populations that practice FGM/C (Isman et al., 2013; Sood et al., 2020). In terms of strategies for eradicating the practice, some researchers have emphasized the importance of involving men. For example, a study conducted in a diasporic context has underscored the value of involving minority migrant men in FGM/C prevention. Male research participants not only expressed ambivalence about continuing the practice, but also showed willingness to reflect on their own role in perpetuating it. They were also open to learning more about the legal implications, medical consequences, and health effects of FGM/C (Axelsson and Strid, 2020). Likewise, a review of program assessments has confirmed the importance of involving men—especially fathers of girls, but also young men who may soon become husbands and fathers (Kohli et al., 2021). Another study has found that, although FGM/C is often described as “women's business,” men's opinions and preferences are also considered. The article in question describes how older women in high-prevalence areas of Senegal will take full responsibility for the practice, but only as a way of protecting male family members from legal repercussions. Women are therefore acutely aware that they are under surveillance and at risk of being charged with violating bans on FGM/C (Shell-Duncan et al., 2021). Finally, these contributions to the literature all highlight the role played by body norms in defining aesthetic and moral acceptability.

However, in diasporic contexts, the social dimension tends to become obscured and women having undergone FGM/C can no longer enjoy any associated benefits (Shell-Duncan et al., 2021). In any case, different approaches have been developed for engaging with migrant populations representing a range of cultures, with a view to encouraging the adoption of more egalitarian gender norms. One study has looked at how messages on FGM/C are received in the context of awareness campaigns. Although research participants reacted positively to the information provided, they also perceived a risk of perpetuating ethnic, gender, and religious stereotypes. For example, certain images were interpreted as suggesting that FGM/C only concerns sub-Saharan women, when the practice is also prevalent in other parts of the world. And whereas some fathers reported feeling singled out by the campaigns, others expressed concern about the association between FGM/C and Islam. They feared that the practice was being presented as a religious issue rather than a cultural one and that Muslims would face stigma and hostility as a result (Salmon et al., 2020). In diasporic contexts, there is certainly a slippery slope from FGM prevention efforts to the adoption of xenophobic or Islamophobic discourses.

Finally, as part of their research in Sudan, Vogt et al. (2016) conducted cultural change “experiments” involving communities that practice FGM/C. In addition to developing a test to measure attitudes toward FGM/C, they produced four movies depicting arguments between members of an extended family who held divergent views on whether the practice should continue. The films were found to have succeeded in changing viewers' outlooks, significantly improving attitudes toward girls who had not undergone FGM/C. The researchers concluded that entertainment can be effective in showcasing “locally discordant views” in a way that helps promote positive cultural change and facilitates cross-cultural exchange. A similar experiment, conducted in distinct socio-cultural contexts across five different sub-Saharan African countries, aimed to measure the effectiveness of a video shown to local populations in promoting family and community dialogue. In this case, the researchers found that the video encouraged joint reflection on FGM/C by close relatives (spouses, members of the same immediate family). And although the study did not find that rates of FGM/C declined directly as a result of the experiment, it did show that critical reflection on the topic can help people to take a step back and reassess whether such traditions should be maintained (Villani et al., 2019b).

## 3. Prospects for the field of social work

The literature review presented above clearly shows the vital importance of initiating discussions on sexuality. Furthermore, addressing the multiple dimensions of sexuality requires a holistic approach to sex education (Charmillot and Jacot-Descombes, 2018), one that avoids medical bias by looking beyond physiological and biological issues. Examining sexuality through a purely medical lens tends to pathologize behavior without addressing its social, political, cultural, and symbolic dimensions. Meanwhile, social work can play a key role in facilitating discussions capable of dispelling myths and challenging beliefs about sexuality—including gendered understandings of sexual and emotional behavior, and intergenerationally transmitted sexual and gender norms—while supporting family-based sex education. Finally, the success of efforts to promote equality is likely to depend on the implementation of policies focused on personal empowerment (i.e., policies that promote a holistic approach to sex education or the enforcement of sexual rights), as well as on the adoption of less stereotypical representations of sexuality in the media and elsewhere in society—all while avoiding the slippery slope to Islamophobic and racist discourses and policies.

## Author contributions

The author confirms being the sole contributor of this work and has approved it for publication.
